# Ethical Challenges in Infant Feeding Research

**DOI:** 10.3390/nu9010059

**Published:** 2017-01-11

**Authors:** Colin Binns, Mi Kyung Lee, Masaharu Kagawa

**Affiliations:** 1 John Curtin Distinguished Emeritus Professor of Public Health, Curtin University, Perth 6845, Australia; 2 Department, School of Health Professions, Murdoch University, Perth 6150, Australia; m.k.lee@murdoch.edu.au; 3 Institute of Nutrition Sciences, Kagawa Nutrition University, Saitama 350-0214, Japan; mskagawa@eiyo.ac.jp

**Keywords:** Infant feeding, research, ethics, consent, breastfeeding, trials

## Abstract

Infants have a complex set of nutrient requirements to meet the demands of their high metabolic rate, growth, and immunological and cognitive development. Infant nutrition lays the foundation for health throughout life. While infant feeding research is essential, it must be conducted to the highest ethical standards. The objective of this paper is to discuss the implications of developments in infant nutrition for the ethics of infant feeding research and the implications for obtaining informed consent. A search was undertaken of the papers in the medical literature using the PubMed, Science Direct, Web of Knowledge, Proquest, and CINAHL databases. From a total of 9303 papers identified, the full text of 87 articles that contained discussion of issues in consent in infant feeding trials were obtained and read and after further screening 42 papers were included in the results and discussion. Recent developments in infant nutrition of significance to ethics assessment include the improved survival of low birth weight infants, increasing evidence of the value of breastfeeding and evidence of the lifelong importance of infant feeding and development in the first 1000 days of life in chronic disease epidemiology. Informed consent is a difficult issue, but should always include information on the value of preserving breastfeeding options. Project monitoring should be cognisant of the long term implications of growth rates and early life nutrition.

## 1. Introduction

Infants are not just small adults. They have a complex set of nutrient requirements and interactions to account for the high metabolic rate, growth, immunological and cognitive development, etc. Infant nutrition lays the foundation for a healthy life. Centuries of observation and research have described the relationship between appropriate nutrition and child growth and survival. In the past century major advances have been made in understanding basic nutrient needs and applying this data to the benefit of humanity and particularly to infants and children. It is appropriate that the collection of original research papers and reviews on infant nutrition is being published in this volume of “Nutrients” at the beginning of the United Nations Decade of Action on Nutrition 2016–2025 [1]. The goals of the decade include increasing rates of exclusive breastfeeding to six months, reducing wasting and stunting and reducing the rates of low birthweight. However the progress in infant nutrition has only served to make the ethical issues around research in this age group even more complex. In this review and discussion paper we will outline progress in infant nutrition and the ethical issues facing infant nutrition researchers and the wider scientific community, particularly the difficulties in defining an adequate consent process. 

### Progress in Infant Nutrition

This year our world will welcome 131 million new infants, but around 5.5 million will die before reaching the age of five years. While this number is still unacceptably high it is only one fifth of the number dying 50 years ago [2]. The global under-5 years mortality rate has fallen by 53% since 1990 to 42.5 per 1000 live births in 2015, still a heavy burden for families to bear. The difference between regions is great with an under-5 years death rate of 98.7 in West Africa compared to 17.1 in Europe. In some individual countries, such as China, Korea, and Vietnam, the rate of decline has been more rapid than the average improvement for the developing world. In the Republic of Korea the under-5 mortality dropped from 330 per 1000 live births to 3.8 between 1950 and 2014, an unrivalled rate of progress. While the improvement has been rapid, the present rates are still far too high and should be seen as a call to further action to improve infant nutrition and health.

One of the most important factors in declining child mortality has been in the improvements made to the food supply and the scientific understanding of the global food supply and nutrient requirements [3,4]. The history of nutrition is a fascinating journey that encompasses a wide spectrum of scientific disciplines from epidemiology and public health to biochemistry and genomics [5]. Many of the advances in nutrition have been driven by clinical necessity as exploring the causation of deficiency syndromes has led to understanding the roles of specific nutrients. Good examples are vitamin C, vitamin A, and thiamin where clinical syndromes led eventually to the understanding of biochemical pathways [6,7,8,9]. During the 19th and 20th centuries the role of nutrients in producing structural, biochemical and behavioural deficiencies was unravelled [10,11]. The bone deformities of children with vitamin D deficiencies have almost gone from the industrialised world and knowledge of iodine and folate has meant that supplementation can reduce the neurological deficiencies they cause [12,13,14,15,16]. In the last half of the 20th century the work of Scrimshaw, the Jelliffes, and Widdowson improved the understanding of the relationships between poor nutrition, growth and infectious diseases, a major factor in the decline of child deaths [17,18,19,20,21].

The simple models of a linear relationship between nutrient intake and function has been replaced with the knowledge that it is often a U-shaped curve that may be made more complex by genomics or interactions with other nutrients [22]. There are many nutrients where a deficiency or an excess of a nutrient, such as vitamin A, can have pathological effects, while amounts consistent with the nutrient reference values (or dietary reference values) result in optimal health outcomes [23,24]. For many infants in vitamin A-deficient regions supplementation has been of considerable benefit. However while vitamin A supplementation in mothers and infants has been widely practised its benefits on a broad scale are still being evaluated [25,26,27,28]. Vitamin D supplementation reduces bone pathology in deficient infants and is recommended in breastfed infants in some countries but, again, the longer-term outcomes of country programs are uncertain [29].

As food supplies have improved and utilisation efficiency has increased with the control of infections, excess of energy intake over expenditure has resulted in our current epidemics of obesity. The double burden of nutrition has made nutrition education so much more complex as we grapple with the difficulty of discussing deficient and excess energy intakes within the same country or even within the same family [30]. 

While probiotics have been in use for many years the complexity of the human microbiome and the relationships of any changes to nutritional status has been the subject of much recent research. In 2007 the National Institutes of Health launched the Human Microbiome Project to stimulate research in an area with potential for human health benefits and much progress has been made in our understanding of this area [31]. Disruption of the human microbiome is now recognised as a potentially important mechanism in the development of obesity in in infants and children [32,33]. The behavioural sciences have always been important in nutrition. New generations are forgetting what “normal” infants and children look like and the image of overweight or obesity has become the new idealised normal [34]. Parents and health professionals have to be re-educated about the importance of growth management as a component of paediatric nutrition. 

One of the most far reaching developments has been our understanding of the relationships between early growth and development and later disease, the Developmental Origins of Health and Disease (DOHAD) hypothesis [35,36,37,38,39]. The structure of health care has been segmented by ages and paediatricians never get to see the cardiac problems of middle age. But this can no longer be the case. The way infants and children are managed has implications for lifelong health, leading to the emphasis on the first 1000 days from conception [40]. If antibiotics are used at birth (and in parts of Asia 100% of mothers receive antibiotics during childbirth) or infant formula is given as the first feed it may disrupt the microbiome with potential lifelong implications for conditions such as obesity or diabetes [41,42,43]. Long term changes to the microbiome may be one mechanism by which early life dietary intake can modify health in later life [44,45].

The development of the knowledge-base of infant nutrition has made many advances in the last decade confirming the supremacy of breastmilk over other sources of infant nutrition. Information is now available on the short- and long-term results of infant feeding decisions making research into infant nutrition very complex as any intervention that diminishes the likelihood of exclusive breastfeeding to six months is likely to be unethical. Once breastfeeding has stopped or been compromised by partial supplementation, it is very difficult, if not impossible, to resume full breastfeeding. The task of those involved in infant nutrition research is to ensure that the highest ethical standards are followed so that breastfeeding is not compromised. 

The ethical issues of infant nutrition research extend into the way that human milk substitutes are sometimes marketed [46,47,48,49]. Infant nutrition is being subjected to an unprecedented amount of advertising to parents and promotion to health professionals and formula sales are increasing [49,50,51]. Parents in the newly-rich countries seek to give their children the best possible start in life. Advertisers often distort advances in science. For example if a nutrient is present in breastmilk the formula companies assume that it will be equally beneficial if added as a pure chemical to a manufactured milk mixture. This ignores the complexity of breastmilk which contains thousands of complex chemicals, living cells, and probiotics [52]. The international news media are quick to draw attention to the existence of any contaminants in breastmilk, ignoring the fact that it is still safer and more beneficial than artificial formula. Professionals involved in human lactation and infant formula research should ensure that their work is not misused in marketing in contravention of the WHO Code [53,54].

Several centuries of infant nutrition research can be summarised in two phrases: “Breast is best” and “Babies were born to breastfeed” [55,56,57]. All international and national infant feeding guidelines reiterate the premier role of breastfeeding, including the benefits of promoting breastmilk for low birth-weight and preterm infants in neonatal intensive care units [58]. The objective of this paper is to discuss the implications of developments in infant nutrition for the ethics of infant feeding research and the implications for obtaining informed consent.

## 2. Materials and Methods

To compile this narrative review a search of databases for papers relevant to infant feeding research ethics was undertaken for publications in the past 25 years. The databases searched were PubMed, Science Direct, Web of Knowledge, Proquest, and CINAHL using combinations of the keywords “Infant Feeding”, “Ethics”, “Trial(s)”, “Research”, “Guidelines”, and “Consent”. Additional searches were made in several relevant journals including the Journal of Human Lactation, Breastfeeding Medicine, Breastfeeding Review, Public Health Ethics, Hastings Review, New England Medical Journal, JAMA, and Pediatrics. The policy documents from selected regulators in the USA, European Union, Canada, and Australia were reviewed. An additional database of infant feeding studies (5846 entries) continuously compiled by the authors was also used. From the potential 9303 papers identified the full text of 87 articles that contained discussion of issues of consent in infant feeding trials were obtained and read. The papers were further assessed and when issues related to the principles of ethical decisions in infant feeding studies were discussed they have been included in this review. Papers were excluded if they were related to adults, children (not infants), or were not directly related to consent in infant feeding trials. A further 45 papers were excluded in this way, leaving 42 papers that were included in the narrative review. 

## 3. Results and Discussion

### 3.1. The Ethics of Infant Nutrition Research

In order for infants to benefit from an improved understanding of nutrition and possible improvements in clinical outcomes research must ultimately be conducted on human subjects, and this means research using infants. The ethics of infant nutritional research are becoming more complex. Ethical approval prior to research commencement is a universal requirement for publication and all of the papers describing trials that were read for this review had received ethical approval. The major principles of medical research have evolved over many centuries and include the minimisation of risk to participants, informed consent, confidentiality, and the right to withdrawal without prejudice. The National Health and Medical Research Council (Australia) states that the values in human research include “respect for human beings, research merit and integrity, justice, and beneficence” and these help to shape the research relationship “as one of trust, mutual responsibility and ethical equality” [59]. The practical implementation of these principles has in the past lagged behind the theory and in the 1950s and 1960s, Pappworth (in the UK and Europe), and Beecher on the opposite side of the Atlantic, drew attention to the violations of ethical research practices occurring on a daily basis [60,61,62,63,64]. In the 19th and early 20th centuries, for example, it was convenient to use children in institutions, invariably from lower socio-economic backgrounds for trials, including smallpox and pertussis vaccination and the transmission of hepatitis and HIV [65,66]. There were many further examples found of research that were clearly unethical on which discussions could be based [67]. The widespread discussion of ethical issues in human research that resulted led to a tightening of research ethics requirements for all research involving humans and the publication of the Helsinki Declaration [68]. Human research ethics committees became mandatory and no journal editor today would knowingly publish a study where there was doubt about the ethics of the study. The International Committee of Medical Journal Editors has built on the Helsinki declaration and has standards for the publication of journal articles that include reference to the ethics of research [69,70]. However, these do not have any special reference to research on infants, other than inclusion as one of the especially dependent groups.

More recently attention has been given to research ethics guidelines that specifically include infants [71]. The Early Nutrition Academy (ENA) and the European Society for Pediatric Gastroenterology, Hepatology, and Nutrition (ESPGHAN) have developed a position statement on infant feeding research [72]. The statement recognises the need for special considerations for infants and breastfeeding mothers. They include an important statement on breastfeeding:

“Breast-feeding is recognised as the optimal feeding choice for healthy infants and should be actively protected and promoted. Conditions of studies performed in breastfeeding women and infants, in particular the strategies for subject recruitment and the interaction with parents and health care professionals, should ensure that there is no interference with breastfeeding [72]”. The statement reiterates in a useful way, the many established principles of human subject research. However there are several issues of infant feeding research ethics that are not mentioned in detail. These include who can give consent, what does informed consent mean in the context of breastfeeding, and how are potential life-long effects of not-breastfeeding to be accounted for. In a review of the standards for intervention trials on feeding of infants, Woodside et al. recommend that “trial duration must be long enough to show changes in the primary outcome measure” [73]. No papers were found which mentioned monitoring to minimise possible long term adverse outcomes such as early signs of obesity.

### 3.2. Breastfeeding and Randomised Controlled Trials

In most clinical research randomised controlled trials are ranked higher than cohort studies and other observational research, but the random allocation of a control group to “non-breastfeeding” is not ethical and usually other study designs must be used. Perhaps the best known study of health outcomes of breastfeeding is the PROBIT trial conducted in Belarus which overcame the ethical issues in a unique way [74]. This was a cluster randomised controlled trial of a breastfeeding promotion intervention, which resulted in higher rates of exclusive breastfeeding in the trial hospitals. As well as analysing the results by hospital (intention to treat analysis), analyses were done where the infants who were exclusively breastfed were compared to those who were breastfed, but not exclusively. Individual breastfeeding status was measured at one month and this could have resulted in misclassification if prelacteal, or other early complementary feeds, had been given. The potential misallocation is important as it is now known that the microbiome can be modified by even short-term formula use. This study design overcame the important ethical issues that would have been involved if infants had been directly allocated to breastfeeding and non-breastfeeding groups, but eliminated a true control group of non-breastfed infants. There are also issues of consent to be considered as some individual choice is lost in cluster randomisation [75]. In the case of PROBIT, the trial is really of a breastfeeding education intervention, but it may be the most practical design for an ethical breastfeeding randomized controlled trial (RCT). However the PROBIT data is also analysed as a comparison of longer and more exclusive breastfeeding versus shorter and less exclusive breastfeeding. In this case the allocation to groups at one month may be a critical distinction since even a short duration of breastfeeding could be protective against certain diseases or adverse outcomes, if compared with no breastfeeding at all, as in the Pima studies [76,77].

### 3.3. Consent in Infant Feeding Trials

Consent in infant feeding studies is a basic ethics consideration. Noel-Weiss discusses studies of lactation and describes how the USA distinguishes between studies that would benefit only the neonate or both the infant and mother (infant mother breastfeeding dyad) [78]. In the former case both parents are required to give consent while the involvement of the mother-infant dyad requires only the consent of the mother [79]. In Canada there are similar requirements and Menon and colleagues have documented the consent process in studies of children in critical care studies [80,81]. One of their concerns was how a lower rate of consent may bias results of the study. The situations they studied raise similar issues to infant feeding studies in neonatal intensive care units and demonstrate the potential conflict between the desire to obtain informed consent and the needs of the research project to obtain a high consent rate to minimise bias. 

In Europe the study must comply with usual ethical principles with an emphasis on potential benefit to the participants and community [82,83]. In Australia the National Health and Medical Research Council oversees the implementation of the National Ethical statement which requires the consent on one parent [59]. There is no reference to the situation where parents have separated and a family court (or equivalent judicial body) mandated joint custody agreement is in place.

Consent implies that it is informed and parents must be given adequate and accurate information. There is no dispute; breastfeeding is best for infants. However, is there an inherent right of an infant to be breastfed? There are some medical contraindications to breastfeeding [84]. There are also the rights of the mother to make an informed decision about breastfeeding. In all cases of infant feeding trials the benefit to an infant to be breastfed should not be interfered with. In preterm infants there is benefit to infants who are breastfed, although some may require additional special feeding regimes. Mothers should be encouraged to continue to express breastmilk to maintain their supply, so that breastfeeding can ultimately be commenced (and continued?).

Screening for metabolic disorders and nutritional deficiencies is now routine in neonatal paediatrics. When a new disease or deficiency is added to a routine screening program, commonly referred to as a pilot study this should be considered to be research and ethics approval and informed consent are required [85].

### 3.4. Beneficence

Beneficence literally means “doing good to others” and in the context of research ethics also includes “non-maleficence”, avoiding doing harm [59]. The National Health and Medical Research Council then concludes this section (4.2.5) “the circumstances in which the research is conducted should provide for the child’s safety, emotional and psychological security, and wellbeing” and (Section 4.2.13) “Before including a child in research, researchers must establish that there is no reason to believe that such participation is contrary to that child’s best interest [59]”.

In light of the research into the disadvantages of not breastfeeding and the advantages of breastfeeding the principle of beneficence means that, wherever possible, an infant should receive breastmilk. Ideally the recommendations of the WHO should be followed: “exclusive breastfeeding for six months followed by continued breastfeeding while complementary foods are also given” [56]. Where this is not possible any breastfeeding is better than none [56]. In any infant nutrition research it is important that there be no interference with the mother’s intention to breastfeed [86].

### 3.5. Monitoring of Infant Feeding Trials

The principle of beneficence extends to longer term risk. Fewtrell describes the change in infant nutrition research from just eliminating nutritional deficiencies to promoting optimal health which then requires a longer term perspective. “This approach necessitates identifying appropriate outcome measures for both safety and efficacy, and also introduces the additional challenges of longer-term follow-up of study participants [87]. There is increasing evidence for early life influences, including nutrition, on later health, the developmental origins of health and disease (DOHAD) [35,36,37,38,39]. The rate of infant growth, too slow or too rapid may be related to later health. 

Research that involves the use of human milk substitutes in both preterm and term infants has used growth and survival as primary objectives for evaluation. To these should also be added longer term objectives including potential for excessive growth (obesity), alterations to and disruptions of the microbiome and longer term chronic disease outcomes (DOHAD). Infant feeding researchers need to be cognisant of possible life-long implications of their research. For example the development of protocols to promote rapid catch up growth in low birth weight infants may possibly be related to increased adult morbidity [88,89].

Further research into relevant clinical markers of long term outcomes is required, but those known to be significant are growth, growth velocity and, perhaps, microbiome alterations. The role of an independent monitoring committee should include checking the rate of individual growth to ensure that it is not too rapid and would increase risk of obesity or later chronic morbidity. “Catch-up growth” of very low birth weight (VLBW) infants is important to ensure survival, but until more is known about long-term risk. A prudent policy is to aim for growth in the middle centile. It is obviously impractical to monitor infant feeding trials for a lifetime for adverse effects, but monitoring of growth rates and signs of early obesity is important. The role and challenges of monitoring committees in clinical trials has been described by De Mets and colleagues [90]. Monitoring of the impact of nutrition interventions on the microbiome may also become advisable as the available technology improves. The principles of ethics applicable to infant feeding studies will need to be kept under review as nutrition science makes further progress.

## 4. Conclusions

A balanced approach to infant nutrition is required. Some infants will require an infant formula and some mothers will choose not to breastfeed. There is no doubt that advances in infant nutrition applied to infant formula have substantially improved infant and child health outcomes. It is essential that this research continues within the constraints of research ethics and the public health imperative of continuing to promote breastfeeding. For infant feeding research involving breastmilk substitutes, the strict implementation of research guidelines is required. As knowledge of the benefits of breastfeeding and the importance of early nutrition, growth, and development on later health, this has increased the responsibility of researchers to strictly apply the ethical principles of beneficence by improving the quality of the information given when obtaining informed consent. This particularly includes the provision of information for informed consent that includes the benefits of breastfeeding and the importance of maintaining lactation. The principle of beneficence would seem to require long-term monitoring of growth and metabolic parameters to minimise any risk to long-term health. In the future, the requirements for ethics approval and longer term monitoring of research outcomes are likely to be strengthened as further advances in research occur.
